# Intestinal metaplasia of the bladder in 89 patients: a study with emphasis on long-term outcome

**DOI:** 10.1186/s12894-016-0142-x

**Published:** 2016-06-07

**Authors:** Zhixiang Xin, Chenhui Zhao, Tao Huang, Zhaohui Zhang, Chenlong Chu, Caifeng Lu, Min Wu, Wenlong Zhou

**Affiliations:** Department of Urology, Rui Jin Hospital Lu Wan Branch, School of Medicine, Shanghai Jiaotong University, No.149, South Chongqing Road, Shanghai, 200020 China; Department of Pathology, Rui Jin Hospital Lu Wan Branch, School of Medicine, Shanghai Jiaotong University, Shanghai, 200020 China

**Keywords:** Intestinal metaplasia, Bladder neoplasm, Cancer risk, Transurethral resection

## Abstract

**Background:**

Intestinal metaplasia of the bladder is an uncommon glandular proliferation. We examined a large series of intestinal metaplasia for the clinicopathological features and discuss the significance of this lesion.

**Methods:**

All cases of intestinal metaplasia diagnosed in our institution between 1990 and 2014 were retrospectively reviewed. Patients with a history of urothelial carcinoma or concurrent adenocarcinoma were excluded. Patient characteristics, pathological features, and follow-up outcomes were obtained.

**Results:**

We identified 89 patients with intestinal metaplasia during this period. Sixty seven were men and 22 were women. Mean age at diagnosis was 57 years (range 23–81). Common presenting complaints included haematuria (73 cases), mucosuria (13 cases), and irritative voiding symptoms (seven cases). The majority of intestinal metaplasias located on or near the trigone (67 cases). Eighty-two patients underwent transurethral resection of their lesions. Partial cystectomy was performed in the remaining seven patients. The mean follow-up of 78 patients was 105 months (range 6–255). One case of bladder adenocarcinoma was indentified 6 months later. The initial histologic findings had revealed intestinal metaplasia with severe dysplasia. Four patients presented recurrence during the follow-up, and this occurred 9, 13, 17 and 24 months after the surgery.

**Conclusions:**

Although intestinal metaplasia can be treated effectively by transurethral resection in most cases, its potential malignancy need to be taken into consideration after the evidence of recurrences and its association with bladder adenocarcinoma. Therefore, it is necessary to perform close surveillance following the surgery, particularly in patients with dysplastic changes.

## Background

Intestinal metaplasia (IM) of the bladder, characterized by the presence of intestinal type epithelium in the bladder, are glandular proliferation that most frequently occur on the bladder trigone [1, 2]. This condition affects men much more commonly than women with an overall estimated incidence of 0.1 to 0.9 % [3, 4]. Most cases of IM are diagnosed in the fifth to sixth decades of life and they are thought to be acquired secondary to persistent irritation and inflammation [1, 5]. Patients have been classically considered to present with hematuria, lower urinary tract symptoms and mucosuria, although many patients instead present with nonspecific urinary complaints. Despite an improved clinical understanding of these uncommon lesions the pathogenesis remains unclear. Proposed etiologies of IM are congenital and acquired causes, including bladder extrophy, long-term catheterization, bladder calculi and neurogenic bladder [1].

The presence of IM as a precursor of adenocarcinoma, a type of bladder cancer with poor prognosis [6], has long been debated. In 1958, Shaw et al. published the first report implicating IM in the development of adenocarcinoma of the bladder [7]. After this research, sporadic case reports have associated IM with bladder adenocarcinoma, prompting consideration of IM as a preneoplastic condition [4, 8, 9]. However, the premalignant nature of IM has been questioned by the following investigators. After reviewing 53 cases, Corica et al. concluded that IM did not seem to be a risk factor for bladder cancer [10], and frequent and long-term follow up was not advocated if a concurrent diagnosis of malignancy was excluded [11]. Such published results have brought conflicting information to the practicing urologist.

To further address this topic, we analyzed the clinicopathological characteristics of 89 cases of IM in our institution and to determine the long-term outcomes in patients with this lesion.

## Methods

**Fig. 1:**
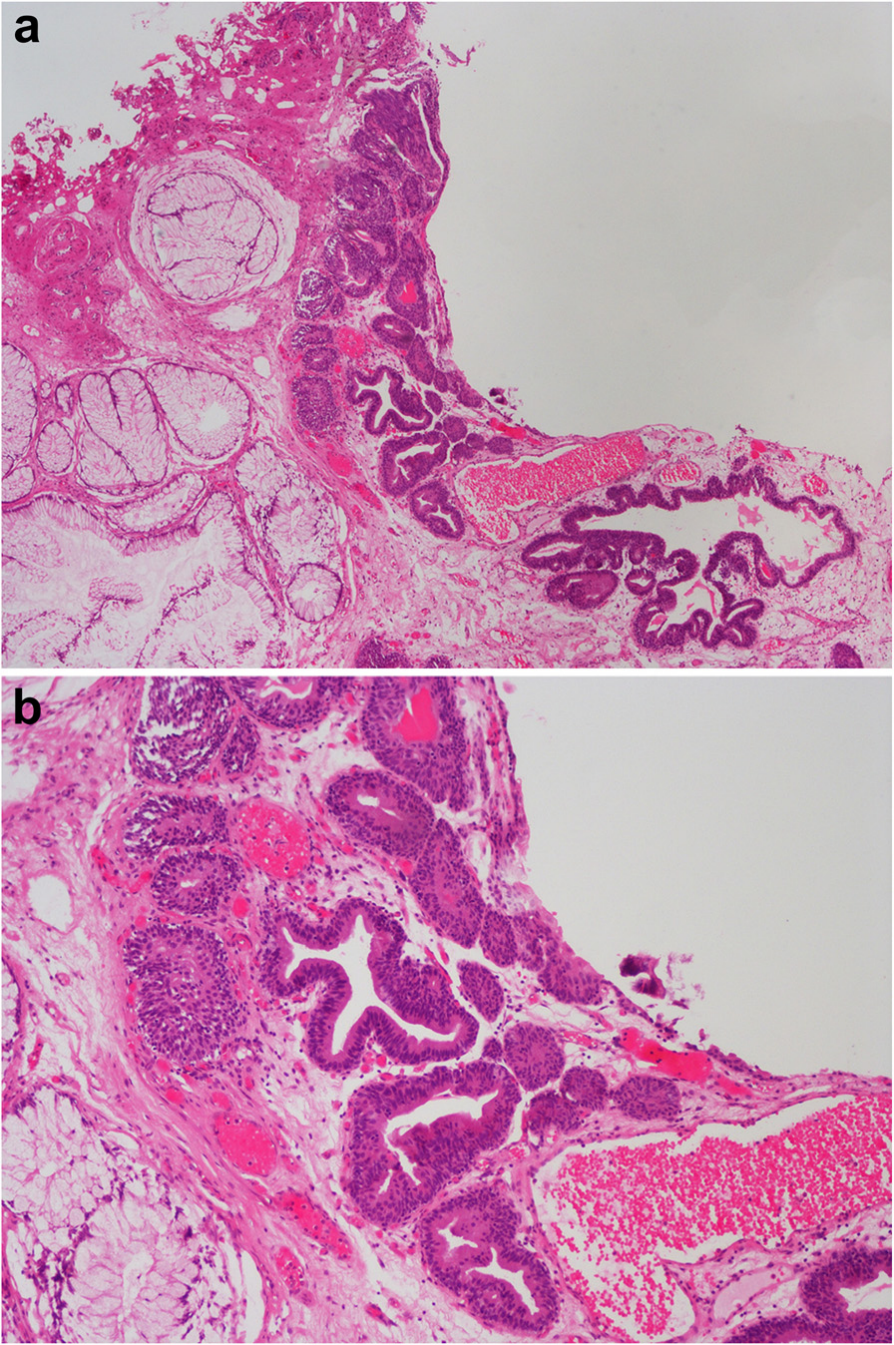
Intestinal metaplasia of the bladder (**a**) The low magnification (× 40) demonstrates numerous orderly distribution of intestinal-like glands in the bladder lamina propria (**b**) On a higher magnification (× 100), the glands are lined by tall columnar epithelial cells without evidence of cytological dysplasia and mitosis

This study was approved by the institutional review board of Rui jin Lu Wan Branch, School of Medicine, Shanghai Jiaotong University. From the bladder tumor database of the Department of Urology, Rui Jin Hospital Lu Wan Branch, all consecutive patients with a diagnosis of bladder IM between 1990 and 2014 were identified. Bladder IM was defined as the presence of columnar-lined epithelium in the bladder mucosa (Fig. 1) [1]. ICD-10 Codes was used in search analysis [12]. Codes that were used for identification of our patient cohort were (1) ‘Malignant neoplasm of bladder’ (C67), (2) ‘Benign neoplasm of Urinary bladder’ (D30.3), (3) ‘Neoplasm of uncertain or unknown behaviour of urinary organs’ (D41), (4) ‘Other cystitis’ (N30.8), (5) ‘Cystitis, unspecified’ (N30.9), (6) ‘Other specified disorders of bladder’ (N32.8), and (7) ‘Bladder disorder, unspecified’ (N32.9). We then manually reviewed all the summary text of pathology reports that meet the criteria, and examined whether the presence of IM or columnar lined bladder was mentioned.

Patients with a baseline diagnosis of bladder adenocarcinoma in IM were excluded, as were patients with other urothelial malignancy, registered prior to, or simultaneously with, the initial diagnosis of IM. The clinical features, including age, sex, presenting symptoms, cystoscopic findings, and follow-up status, were obtained from the medical records and pathology reports.

The follow-up period was defined as the time between the date of the first cystoscopy with biopsy at which the diagnosis of bladder IM was made and the date of the last surveillance cystoscopy. No mandatory surveillance protocol was followed during the study period, but the practice in our unit was to perform cystoscopy at an interval of 3 months during the first year after the surgery followed by yearly cystoscopy.

In all patients included for this study, histological slides (hematoxylin and eosin staining) were retrieved and microscopically reviewed by two pathologists. While reviewing the histological slides, the pathologists were unaware of the follow-up data.

## Results

Among 27,424 patients registered in our bladder tumor database, we identified 98 patients diagnosis with IM between 1990 and 2014, resulting in an incidence of 0.26 % in bladder tumor population. A total of 9 patients were excluded from the analysis because they had concurrent carcinoma at baseline. This left 89 patients for analysis, who were followed up to 21 years. Of this cohort, 67 (75 %) were men and 22 (25 %) were women, with a mean age of 57 years. The peak incidence of diagnosis was in the fifth and sixth decades of life. The clinicopathological characteristics of the patients included are summarized in Table 1.

**Table 1:** Characteristics of the 89 patients with intestinal metaplasia

Characteristics	No. (%)
Sex	
Male	67 (75)
Female	22 (25)
Age, y	57
Follow-up, mo	(Range 23–81)
	105
	(Range 6–255)
Symptom	
Haematuria	73 (82)
LUTS	15 (17)
Mucosuria	7 (8)
Multiple symptoms	11 (12)
AUR	3 (3)
Others	2 (2)
N.A	2 (2)
Tumor location	
Trigone	67 (75)
Dome	2 (2)
Posterior wall	8 (9)
Lateral wall	12 (14)
Left	5 (6)
Right	7 (8)
Treatment	
Transurethral resection	82 (92)
Partial cystectomy	7 (8)
Total case(s) of bladder adenocarcinoma	1 (1)
Total case(s) of recurrent intestinal metaplasia	4 (4)
Died of unrelated disease	4 (4)
Died of disease	0 (0)

*Abbreviation*: *LUTS* lower urinary tract symptoms, *AUR* acute urinary retention, *N.A* not available

The most common presenting complaint was gross or microscopic haematuria, seen in 73 cases (82 %), followed by irritative voiding symptoms (13 cases) and mucosuria (seven cases). Other presenting symptoms included dysuria (two cases), acute urinary retention (three cases), and suprapubic pain (two cases). Eleven patients had more than one of these symptoms. The presenting symptoms of the remaining two patients were not available.

Of the 89 patients, 64 (71 %) had associated with conditions predisposing to the stasis of urine or exposure to chronic irritants such as bladder outlet obstruction (12 cases), chronic urinary tract infections (UTI) (29 cases), bladder stones (15 cases), and neurogenic bladder (eight cases).

The majority of IMs were located on or near the trigone (67 cases or 75 %), and the remaining lesions were found on the dome (two cases), posterior wall (eight cases), and lateral wall (12 cases, 5 on the left and 7 on the right). Cystoscopically, most lesions were described as flat and polypoid masses or papillary lesions with gelatinous surfaces. In 24 cases, multifocal lesions were noted. Eighty-two patients underwent transurethral resection (TUR) of their lesions. Partial cystectomy was performed in the remaining seven patients including two patients with transplantation of ureter.

Cystoscopic follow-up was conducted in 78 patients (88 %). Five patients had only clinical follow-up, and 6 patients had no additional follow-up in our hospital. The mean period of follow-up was 105 months (range from 6 to 255 months). At review, 36 patients (40 %) had follow-up for more 10 years. Four patients died of unrelated diseases during the follow-up period (one of gastric cancer, one of pneumonia, one of myocardial infarction, and one of brain hemorrhage).

Of the 78 patients, four patients (5 %) developed recurrent IM, and this occurred 9, 13, 17 and 24 months after resection of the initial lesion, respectively (Table 2). In the first case IM developed in two places 9 months after TUR, and IM recurred in two places 1 year 3 months after the surgery. In this example IM recurred two times thereafter and the patient currently undergoes follow-up observation. Similar to the first case, in the second case IM recurred 2 times on the trigone after the surgery. However, this patient refused to take further re-TUR. In the third case TUR was performed due to the development of IM on the left lateral wall, and IM recurred near the posterior wall 1 year 5 months after surgery. At this time, 7 years 2 months after the initial recurrence, this case remains free from further recurrence. In the forth case TUR was performed for the development of IM on the trigone, and IM developed laterally in the right ureteral orifice 2 years later. Unfortunately, IM recurred 9 months later and the patient required re-TUR.

**Table 2:** Clinical details of the four patients that subsequently developed recurrence following the surgery

Pat. No.	Gender	Age at surgery, yr	Onset Site	Recurrence Site	Interval Before Recurrence	Outcome
1	Male	57	Bladder trigone & Left lateral wall	Bladder trigone & Left lateral wall	9 months	Recur 15 months later
2	Male	73	Bladder trigone	Bladder trigone	13 months	Recur 11 months later
3	Male	64	Left lateral wall	Posterior wall	17 months	Remains free from further recurrence
4	Male	62	Bladder trigone	Laterally in the right ureteral orifice	24 months	Recur 9 months later

Of note, one patient (1 %) developed subsequent bladder adenocarcinoma. The patient was a 62-year-old male. He had presented with macroscopic hematuria, and his lesion was extensive but mainly located on the trigone. Although the immediate repeat cystoscopy after TUR was normal, tumor had developed on the trigone 6 months later. Histologic review of the originally diagnosed IM disclosed glandular structures lined by dysplastic columnar cells.

## Discussion

Although IM are reported to occur in 0.1 to 0.9 % of the bladder population, the true incidence is difficult to determine due to the vague, nonspecific symptoms often associated with this condition [3, 4]. In this study, we studied 89 patients from 27,424 bladder tumor patients with long-term follow-up (mean 105 months) after the initial diagnosis of IM. To the best our knowledge, this represents the largest series of IM cases described in a single institution. In the current series most patients reported symptoms of haematuria, irritative voiding symptoms and mucosuria in the fifth to sixth decade of life, which is comparable to that in prior studies [10, 11]. However, the frequent finding of gross or microscopic haematuria in 82 % of our patients is much higher than the rates previously reported in patients with IM [10].

Although the etiology of IM is not well characterized, many cases are thought to represent an acquired condition that occur secondary to long-standing injurious stimulus [1, 5]. More than half of the patients in our study had associated conditions predisposing to the stasis of urine or exposure to chronic irritants. Persistent inflammation of the bladder has been proposed to lead to adaptation and subsequently transformation of the urothelium, resulting in the development of glandular metaplasia [13]. Of the patients in our series 33 % had a history of chronic UTI, suggesting a possible association between this finding and an IM in a large proportion.

Grossly, IM can be found anywhere in the bladder but usually arises from the trigone [2]. In our cases, 75 % of the IM developed on or near the triangle region, which is almost consistent with the reports of previous investigators [2]. It is important to note that over one-fourth of the cases are multifocal, highlighting the entire urothelium needs to be evaluated if a lesion is found.

In theory, the urothelium may return to a normal pattern of differentiation, if the stimulus that caused metaplasia is removed or ceased. However, patients often require surgical intervention due to ongoing clinical symptoms or concern about dysplastic changes. Because IM has no tendency toward infiltration, and under most circumstances is superficial [1], nearly all cases in our series were treated by TUR. However, difficulty in differentiating extensive IM from adenocarcinoma and occasional coexistence of IM and carcinoma make cystectomy unavoidable in some cases. In our cases, seven patients underwent partial cystectomy to remove the lesions.

The significance of IM of the bladder has been the subject of numerous debates [1, 5, 13–15]. On the one hand, numerous of molecular changes have been demonstrated to be present in the metaplastic changes which indicate that IM may constitute a putative precursor lesion of adenocarcinoma. By applying the fluorescent in situ hybridization, Morton et al. provided evidence of significant telomere shortening in IM compared with telomere length in adjacent normal urothelial cells. In addition, chromosomal abnormalities associated with urothelial carcinoma were shown to be present in a subset of IM. The authors concluded that these findings suggest a premalignant potential of IM [16]. Bryan et al. investigated the role of tumor necrosis factor-alpha and the adherens junction component beta-catenin in IM, and found nuclear localization of the latter in IM. Since nuclear localization of beta-catenin is also seen in the Barrett metaplasia of the esophagus, which is a preneoplastic condition [17], the authors concluded that bladder IM may have the same potential to progress to malignancy [18].

On the other hand, to date only six cases of carcinoma arising in IM have been reported [4, 7–9, 11, 19]. It is noteworthy that the malignancies in the IM appear not to be unique, but one of the standard bladder cancers, ie adenocarcinoma or transitional cell carcinoma, with the majority of tumors reported being adenocarcinomas. However, many documented cases were derived from case reports and have been criticized to have a history of a prior or concurrent carcinoma. Furthermore, in a clinical investigation of 53 patients with IM, Corica et al. found that none of the patients developed bladder carcinoma after a median follow-up of 13 years. The authors concluded that IM is not a strong risk factor for bladder carcinoma [10]. Smith et al. studied 12 cases of IM and found that only one patient developed an urothelial carcinoma 3 months after resection of an IM; they concluded that IM does not seem to increase the future risk of bladder malignancy and surveillance cystoscopy is not recommended in such patients [11].

In our study we identified carcinoma arising within 1 % of IM and in this case in our series it represented adenocarcinoma. The result is in contrast to the findings in previous studies [10, 11]. Several explanations may account for this discrepancy. First, our cohort includes the largest number of IM patients to date with long-term follow-up. This obviously differs from previous small study with relatively short follow-up, which may not have adequate power to capture the incidence of events [11]. Second, in Corica et al. study, most of recruited patients were children rather than adults in our series [10]. Metaplastic cells in children with extrophy might occur at early stage in the replicative cycle and require decades to progress to carcinoma [20]. Third, all patients with extrophy have been underwent surgery to reconstruct the bladder. It is reasonable to consider that repair of the extrophy likely removed the stimulus and delay or reverse the process of carcinogenesis [10].

Although we could not rule out the possibility that development within 6 months of a carcinoma in our study was because an overlook at the time of diagnosis, this single case highlights several important clinical features. Extensive lesion was present in the initial cystoscopic findings but, more importantly, the histologic examination indicated severe dysplastic changes. Recently, Gordetsky et al. reported the histologic details and follow-up of patients with dysplasia. With a total of 20 cases included, the authors concluded that dysplastic changes are significantly associated with concurrent adenocarcinoma and suggested that patients with IM exhibiting this feature should undergo close follow-up [19].

This might raise the question of whether the presence of dysplasia is an indicator for the development of subsequent adenocarcinoma. The metaplasia-dysplasia-adenocarcinoma sequence is a widely recognized event in several epithelial tissues including esophagus and gastric [17, 21]. In supporting this sequence in bladder IM, Srivastava et al. demonstrated positive expression and allelic imbalance of TP53 and loss of heterozygosity for D2S123 in glandular dysplastic foci but not in IM. The authors therefore suggested that the presence of dysplasia might be the early changes in the stepwise progression to adenocarcinoma [22]. Unfortunately, our data could not provide sufficient evidence to answer this question. More studies with patients with dysplasia are needed.

Implication for malignant potential of the IM might also be obtained in the number of recurrences, which has been estimated to be around 6 % in previous study [10]. Of 78 patients for whom follow-up was presented in our study, 4 (5.0 %) were reported to have had recurrent lesions after a follow-up of 9 to 24 months. Although the rates are lower than in transitional cell carcinoma, in three cases the IM recurred two times. In fact, when cases of recurrence are examined IM develops near the origin site in many at the time of recurrence, and cases with a history of chronic UTI before the development of IM are seen frequently.

Our study has some methodologic factors that might affect the accuracy of our estimates. First, it was a retrospective study of a limited number of patients. The patients were not followed up uniformly at regular intervals, and the progression and recurrence may be higher if patients were followed more closely or regularly. Second, immunohistochemical staining was not routinely undertaken during the study period. In contrast to other glandular lesions, IM commonly expresses staining for CDX2 and CK20, which is often seen in colonic mucosa [23]. Third, All lesions were surgical removed before the start of follow-up. Hence, there was no residual lesion in the bladder and all patients could be regarded to have normal urothelium at the beginning of follow-up. Therefore, the nature history of IM remains to be elucidated.

## Conclusions

While intestinal metaplasia of the bladder are uncommon, they are not rare in the male patients with chronic urinary tract infection. The question of whether intestinal metaplasia is a premalignant condition continues to be a matter of debate. Although treatment such as transurethral resection appears to be effective in most cases, approximately 5 % of patients demonstrate postoperative recurrence or progress to malignancy. Since the natural history of intestinal metaplasia is unknown, close surveillance following the surgery is necessary, particularly in patients with dysplastic changes.

## Abbreviations

IM, intestinal metaplasia; TUR, transurethral resection; UTI, urinary tract infection.
